# Protocol for a scoping review of neonatal emollient therapy and massage practices throughout sub-Saharan Africa

**DOI:** 10.12688/gatesopenres.13359.1

**Published:** 2021-08-24

**Authors:** Keona J.H. Blanks, Milton W. Musaba, Lily Ren, Kathy Burgoine, David Mukunya, Andrew Clarke, Sarah Williams, Tewodros Gebremichael, Peter Waiswa, Gary L. Darmstadt

**Affiliations:** 1 Stanford University, 450 Serra Mall, Stanford, CA, 94305, USA; 2 Department of Obstetrics and Gynaecology, Busitema University Faculty of Health Sciences, Pallisa, Mbale, PO Box 1460, Uganda; 3 Lane Medical Library & Knowledge Management Center, Stanford Medicine, Stanford University, 300 Pasteur Drive, L109, Stanford, CA, 94305, USA; 4 Mbale Clinical Research Institute, Plot 29, 33 Pallisa, Mbale, Uganda; 5 Busitema University Faculty of Health Sciences, Pallisa, Mbale, PO Box 1460, Uganda; 6 Sanyu Africa Research Institute, Mbale, PO Box 2190, Uganda; 7 Global Programs, Save the Children UK, 1 St John's Ln, London, EC1M 4AR, UK; 8 Makerere University, University Rd, Kampala, Uganda; 9 Department of Pediatrics, Stanford University School of Medicine, 1701 Page Mill Road, Palo Alto, Stanford, CA, 94304, USA

**Keywords:** Neonatology, Emollient, Massage, Bathing, Africa, Low- and middle-income countries

## Abstract

**Background:** Serious infections and other complications from very low birth weight and prematurity are the leading causes of death for neonates worldwide. Infections partly result from the compromised skin barrier function in preterm neonates. Optimal skin care practices for neonates, especially in settings with limited access to adequate hygienic conditions, hold potential to reduce infection and avoid these preventable preterm neonatal deaths. The purpose of this protocol is to support a scoping review of neonatal skin care, emollient therapy and massage practices throughout sub-Saharan Africa.

**Protocol: **The proposed review will follow a methodological framework consisting of the following five steps: (i) identifying the research question, (ii) identifying relevant studies, (iii) selection of eligible studies, (iv) charting the data, and (v) collating and summarizing the results. In addition, we will reflect on the implications of the findings for the feasibility and design of randomized controlled trials to examine the impact of emollient therapy on survival, growth, infection and neurodevelopment of very low birth weight infants in sub-Saharan Africa. We will search domestic and international databases for literature published in English between January 1, 2000, and July 12, 2021. Articles will be chosen based on standardized inclusion criteria. The primary criteria for inclusion will be a report on skin care practices administered to neonates in Africa.

**Conclusions: **Documentation of common neonatal skin care practices throughout Africa has the potential to highlight opportunities for skin care intervention and future research on neonatal skin care practices in sub-Saharan Africa, and support the development of future emollient intervention trials for preterm and low birthweight neonates in low- and middle-income countries.

## Introduction

The neonatal mortality rate in sub-Saharan Africa is the highest in the world, estimated at 27 deaths per 1,000 live births ^1^. Worldwide, 98% of all neonatal deaths occur in low- and middle-income countries (LMICs) and are largely ascribed to serious infections, complications from prematurity and birth asphyxia ^2^. Compromised skin barrier function is an important factor in the complications associated with preterm birth ^3^. Lacking in vernix, a protective antimicrobial layer, the skin barrier of preterm infants is functionally compromised and has heightened susceptibility to injury, leading to increased risk for invasive infection and death ^4^. Compounding the issue are maternal malnutrition, neonatal growth restriction and virulent pathogens that pervade unsterile environments in low-resource settings and potentially impact newborn infants at population-level ^5, 6^. Even in hospitals, overcrowding, limited staffing, unreliable water supply, lack of alcohol-based hand wash and lack of bleach and soap for basic cleaning of equipment further exacerbate poor sterilization and disinfection practices.

Topical emollient therapy to the skin of preterm infants is a nascent intervention for neonatal care in LMICs. Emerging evidence suggests that emollient therapy promotes postnatal growth and reduces hospital-acquired infection and has potential to reduce mortality and enhance neurodevelopmental outcome ^7– 9^. While application of oils and other products to the newborn infant is a widespread cultural practice in South Asia, less is known about this behavior in sub-Saharan Africa ^10^. There have likewise been few reports of the effects of emollient therapy on newborn health in sub-Saharan Africa ^11, 12^.

The main objective for the proposed scoping review is to create an accurate depiction of newborn skin care practices in Africa, and to illuminate opportunities for intervention. This review will address the questions of “what are the common newborn skin care practices throughout Africa, with a focus on bathing, the application of oils and other products, and massage?” and “what is the reported impact of newborn skin care practices throughout Africa, for example the impact of emollient therapy on newborn postnatal survival, growth, infection and neurodevelopment?” This information would be critical for researchers planning effective intervention trials in Africa.

## Protocol

This protocol is reported in line with the Preferred Reporting Items for Systematic review and Meta-Analysis Protocols (PRISMA-P) checklist ^13^.

### Scoping review

This protocol is for a scoping review of literature reporting on common neonatal skin care practices throughout sub-Saharan Africa, with a focus on bathing, the application of oils and other products, and massage. A scoping review method was selected for its aims to delineate and identify gaps in available evidence on the area of focus. The proposed review will follow a methodological framework proposed by Arksey and O’Malley ^14^. The framework consists of the following five steps: (i) identifying the research question, (ii) identifying relevant studies, (iii) selection of eligible studies, (iv) charting the data, and (v) collating and summarizing the results. Quality appraisal will not be done as this review aims to map all research activities in this field.

### Identifying the research question

The main research questions are “what are the common newborn skin care practices throughout sub-Saharan Africa, with a focus on bathing, the application of oils and other products, and massage?” and “what is the reported impact of newborn skin care practices throughout Africa, for example the impact of emollient therapy on survival, growth, infection and neurodevelopment?”

This study will use a series of guiding queries to align the study selection with the research question (see below, Charting the data). Since most of the studies relevant to this review are qualitative or observational in design (interviews, focus group discussions, and surveys), this approach was selected in lieu of the Population, Exposure, Comparator, and Outcomes (PECO) framework to allow for the inclusion of these studies. Randomized control trials (RCTs) and quasi experimental clinical trials will also be aligned in this manner.

### Identifying relevant studies

A search will be conducted for published and unpublished (grey) literature on the research question in the following electronic databases: PubMed, Scopus (Elsevier), Embase, Web of Science (Clarivate Analytics), and PsycINFO (Ovid). Sources of grey literature will include: OpenGrey and GreyNet. Studies published prior to July 2021 that have the keywords or Medical Subject Headings (MeSH) terms delineated in the search strategy ( Table 1) will be identified. The search strategy will be piloted to check the appropriateness of keywords and databases. A hand (“snowballing”) search will also be conducted of the references of the included studies and websites such as the World Health Organization (WHO) to identify potentially relevant literature. Grey literature will also be identified through direct queries to authors of included literature to explore whether they are aware of unpublished literature on the topic

**Table 1.** PubMed search strategy for a scoping review of neonatal emollient therapy and massage practices throughout sub-Saharan Africa.

SearchActionsDetailsQueryResults Search: **#4 NOT #5** Filters: **from 2000 - 2021** 3,48516:16:43#21 Search: **#4 NOT #5** 216:16:07#5 Search: **"animals"[mesh] NOT ("animals"[mesh] AND "humans"[mesh])** 4,843,98816:15:38#4 Search: **#1 AND #2 AND #3** Search: "Infant, Newborn"[Mesh] or "Infant Care"[Mesh] or infant* or neonat* or newborn* or "new born*" or "Child Rearing"[mesh] or "child rear*"[tw] or childrear*[tw] or childbear*[tw] or "Postpartum Period"[Mesh] or "postpartum"[tw] 1,678,41916:01:31#2 Search: "africa"[Mesh] or "africa*"[tw] or "sudan*"[tw] or "western sahara*"[tw] or "algeria*"[tw] or "egypt*"[tw] or "libya*"[tw] or "morocc*"[tw] or "tunisia*"[tw] or "Cairo"[tw] or "Rabat"[tw] or "Casablanca"[tw] or "Tripoli"[tw] or "Algiers"[tw] or "Fes"[tw] or "Marrakesh"[tw] or "Tunis"[tw] or "Carthage"[tw] or "Tangier"[tw] or "Kairouan"[tw] or "Essaouira"[tw] or "Luxor"[tw] or "Bizerte"[tw] or "El Aaiun"[tw] or "Sousse"[tw] or "Oran"[tw] or "Annaba"[tw] or "Constantine"[tw] or "Biskra"[tw] or "Chefchaoouen"[tw] or "Skikda"[tw] or "Sharm El Sheikh"[tw] or "Volubilis"[tw] or "El Oued"[tw] or "Meknes"[tw] or "Hippo Regius"[tw] or "Djemila"[tw] or "Sfax"[tw] or "Tataouine"[tw] or "port Said"[tw] or "Ait Benhaddou"[tw] or "Benghazi"[tw] or "Juba"[tw] or "Tamanrasette"[tw] or "merzouga"[tw] or "El Djem"[tw] or "oujda"[tw] or "Matmata"[tw] or "Ghat"[tw] or "Tabessa"[tw] or "Giza"[tw] or "Marj"[tw] or "Ifrane"[tw] or "Agadir"[tw] or "Tetouan"[tw] or "Shubra El Kheima"[tw] or "Tobruk"[tw] or "Khartoum"[tw] or "Nyala"[tw] or "Kassala"[tw] or "Ubayyid"[tw] or "Kosti"[tw] or "Wad Madani"[tw] or "Qadarif"[tw] or "Al-Fashir"[tw] or "Daein"[tw] or "Damazin"[tw] or "Geneina"[tw] or "Merowe"[tw] rr "Sahara"[tw] or "British Indian Ocean Territory"[tw] or "Burundi*"[tw] or "Comoros"[tw] or "Djibouti*"[tw] or "Eritrea*"[tw] or "Ethiopia*"[tw] or "Kenya*"[tw] or "Madagascar"[tw] or "Malawi"[tw] or "Mauritius"[tw] or "Mayotte"[tw] or "Mozambique"[tw] or "Reunion"[tw] OR "Rwanda*"[tw] or "Seychelles"[tw] or "Somalia*"[tw] or "Sudan*"[tw] or "Tanzania*"[tw] or "Uganda*"[tw] or "Zambia"[tw] or "Zimbabwe"[tw] or "Crozet Islands"[tw] or "Iles Crozet"[tw] or "Scattered Islands"[tw] or "Iles Eparses"[tw] or "Addis Ababa"[tw] or "Asmara"[tw] or "Anananarivo"[tw] or "Arusha"[tw] or "Axum"[tw] or "Bahir Dar"[tw] or "Berbera"[tw] or "Bulawayo"[tw] or "Dese"[tw] or "Eldoret"[tw] or "Garissa"[tw] or "Geita"[tw] or "Gondar"[tw] or "Great Rift Valley"[tw] or "Hargeisa"[tw] or "Hargeysa"[tw] or "Hola"[tw] or "Jinja"[tw] or "Iringa"[tw] or "Kigoma"[tw] or "Jimma"[tw] or "Korogwe"[tw] or "Nairobi"[tw] or "Dar es Salaam"[tw] or "Mombasa"[tw] or "Mogadishu"[tw] or "Dodomoa"[tw] or "Bujumbura"[tw] or "Mbeya"[tw] or "Lusaka"[tw] or "Harare"[tw] or "Kakamega"[tw] or "Kampala"[tw] or "Kigali"[tw] or "Kire Dawa"[tw] or "Kikuyu"[tw] or "Kisumu"[tw] or "Kitale"[tw] or "Kitui"[tw] or "Lilongwe"[tw] or "Lake Victoria"[tw] or "Lake Tanganyika"[tw] or "Lamu"[tw] or "Lodwar"[tw] or "Lokichogio"[tw] or "Malindi"[tw] or "Machakos"[tw] or "Marka"[tw] or "Machakos"[tw] or "Maputo"[tw] or "Maralal"[tw] or "Mekele"[tw] or "Meru"[tw] or "Musoma"[tw] or "Mtwara"[tw] or "Mumias"[tw] or "Moshi"[tw] or "Moroni"[tw] or "Morogoro"[tw] or "Mwanza"[tw] or "Naivasha"[tw] or "Nanyuki"[tw] or "Nakuru "[tw] or "Namanga"[tw] or "Nyeri"[tw] or "Port Louis"[tw] or "Puntland*"[tw] or "Nyahururu"[tw] or "Kismayo"[tw] or "Ruiru"[tw] or "Rwenzori Mountains"[tw] or "Sinyanga"[tw] or "Songea"[tw] or "tanga"[tw] or "Tabora"[tw] or "voi"[tw] or "webuye"[tw] or "Zanzibar"[tw] or "Adiharush"[tw] or "Ali-Addeh"[tw] or "Alinjugur"[tw] or "Buramino"[tw] or "Dadaab"[tw] or "Dagahaley"[tw] or "Dollo Ado"[tw] or "Fugnido"[tw] or "Hagadera"[tw] or "Hilaweyn"[tw] or "Ifo"[tw] or "Kakuma"[tw] or "Kambioos"[tw] or "Kayaka II"[tw] or "Kobe"[tw] or "Kyangwali Nakivale"[tw] or "Nyarugusu"[tw] or "Wad Sherife"[tw] or "Bokolmanyo"[tw] or "Melkadida"[tw] or "Rwamanja"[tw] or "benin*"[tw] or "burkina fas"[tw] or "cape verd*"[tw] or "cabo verd*"[tw] or "ivory coast"[tw] or "cote divoire*"[tw] or "gambia*"[tw] or "ghana*"[tw] or "guinea*"[tw] or "bissau"[tw] or "liberia map"[tw] or "malian"[tw] or "mauritania*"[tw] or "nigeria*"[tw] or "senegal*"[tw] or "sierra leon*"[tw] or "togo*"[tw] or "Lagos"[tw] or "Accra"[tw] or "Abidjan"[tw] or "Dakar"[tw] or "Abobo"[tw] or "Abuja"[tw] or "Freetown"[tw] or "Ouagadougou"[tw] or "Conakry"[tw] or "Lome"[tw] or "Bamako"[tw] or "Cotonou"[tw] or "Kumasi"[tw] or "Monrovia"[tw] or "Ibadan"[tw] or "Kano"[tw] or "Port harcourt"[tw] or "Benin City"[tw] or "Porto Novo"[tw] or "Niamey"[tw] or "Yamoussoukro"[tw] or "Banjul"[tw] or "Timbuktu"[tw] or "Djenne"[tw] or "Abomeyu"[tw] or "Zaria"[tw] or "Tamale"[tw] or "Jos"[tw] or "Cape Coast"[tw] or "Maidugul"[tw] or "Aba"[tw] or "Gao"[tw] or "Calabar"[tw] or "Warri"[tw] or "Maiduguri"[tw] or "Bobo Dioulasso"[tw] or "Parakou"[tw] or "Djougou"[tw] or "Bohicon"[tw] or "Sekondi Takoradi"[tw] or "Sunyani"[tw] or "Obuasi"[tw] or "Teshie"[tw] or "Tema"[tw] or "Sikasso"[tw] or "Kalabankoro"[tw] or "Nouakchott"[tw] or "Dakhlet Nouadhibou"[tw] or "Benin City"[tw] or "Port Harcourt"[tw] or "Ilorin"[tw] or "Kaduna"[tw] or "Enugu"[tw] or "Ikorodu"[tw] or "Onitsha"[tw] or "Bauchi"[tw] or "Akure"[tw] or "Abeokuta"[tw] or "Sokoto"[tw] or "Bouake"[tw] or "Makeni"[tw] or "Kaduan"[tw] or "Sosgbo"[tw] or "Osogbo"[tw] or "Gombe"[tw] or "Ilesa"[tw] or "Badagry"[tw] or "makurdi"[tw] or "Sagamu"[tw] or "Iseyin"[tw] or "obbomosho"[tw] or "Awka"[tw] or "Ado Ekiti"[tw] or "Nsukka"[tw] or "Ikeja"[tw] or "Katsina"[tw] or "Okene"[tw] or "Lafia"[tw] or "Minna"[tw] or "Ondo city"[tw] or "Umuahia"[tw] or "Calabar"[tw] or "Yola"[tw] or "Pikine"[tw] or "Touba"[tw] or "Thies Nones"[tw] or "Saint Louis"[tw] or "Kolak"[tw] or "Ziguinch"[tw] or "San Pedro"[tw] or "Bandama"[tw] or "Daloa"[tw] or "Owerri"[tw] or "Kandi"[tw] or "Ifi"[tw] or "Dakar"[tw] or "Ogbomosho"[tw] or "Divo"[tw] or "Korhogo"[tw] or "angola"[tw] or "cameroon*"[tw] or "chad"[tw] or "tchad"[tw] or "congo*"[tw] or "DRC"[tw] or "equatorial guinea*"[tw] or "gabon*"[tw] or "Sao Tome"[tw] or "Principe"[tw] or "Luanda"[tw] or "lobito"[tw] or "kuito"[tw] or "huambo"[tw] or "Malanje"[tw] or "Douala"[tw] or "Yaounde"[tw] or "Bamenda"[tw] or "Garoua of Bafoussam"[tw] or "Nganoundere"[tw] or "Maroua"[tw] or "Kouosseri"[tw] or "Buena"[tw] or "Kumba"[tw] or "N Djamena"[tw] or "Moundou"[tw] or "Bangui"[tw] or "Bimbo"[tw] or "Brazzaville"[tw] or "Point Noire"[tw] or "Kinshasa"[tw] or "Lubumbashi"[tw] or "Leopoldville"[tw] or "Elizabethville"[tw] or "Mbuji Mayi"[tw] or "Bakwanga"[tw] or "Bukavu"[tw] or "Costermansville"[tw] or "Kananga"[tw] or "Luluabourg"[tw] or "Kisangani"[tw] or "Stanleyville"[tw] or "Tshikapa"[tw] or "Koalwezi"[tw] or "Likasi"[tw] or "Jadotville"[tw] or "Goma"[tw] or "Kikwit"[tw] or "Uvira"[tw] or "Bunia"[tw] or "Mbandaka"[tw] or "Coquilhatville"[tw] or "Matadi"[tw] or "Butembo"[tw] or "Kabinda"[tw] or "Mwene Ditu"[tw] or "Isiro"[tw] or "Paulis"[tw] or "Boma"[tw] or "Kindu"[tw] or "Bata"[tw] or "Malabo"[tw] or "Libreville"[tw] or "botswana*"[tw] or "lesotho*"[tw] or "malawi*"[tw] or "mozambiq*"[tw] or "namibia*"[tw] or "swaziland"[tw] or "zambia*"[tw] or "zimbabwe"[tw] or "Zulu"[tw] or "Tsonga"[tw] or "Xhosa"[tw] or "Swazi"[tw] or "Ndebele"[tw] or "Tswana"[tw] or "Sotho"[tw] or "Shona people"[tw] or "BaLunda"[tw] or "Mbundu"[tw] or "Ovimbundu"[tw] or "Chaga"[tw] or "Sukuma"[tw] or "Pretoria"[tw] or "Cape Town"[tw] or "Johannesburg"[tw] or "Durban"[tw] or "Port Elizabeth"[tw] or "Bloemfontein"[tw] or "Windhoek"[tw] or "Maseru"[tw] or "Pietermaritz"[tw] or "Kimberley"[tw] or "Nespruit"[tw] or "Soweto"[tw] or "Polokwane"[tw] or "Limpopo"[tw] or "Rustenburg"[tw] or "Mahikeng"[tw] or "Oudtshroom"[tw] or "Stellenbosch"[tw] or "Paarl"[tw] or "Gaborone"[tw] or "Luanda"[tw] or "Cabinda"[tw] or "Huambo"[tw] or "Lubango"[tw] or "Kuit"[tw] or "Malanje"[tw] or "Lobito"[tw] or "Lilongwe"[tw] or "Blantyre"[tw] or "Mzuzu"[tw] or "Maputo"[tw] or "Matola"[tw] or "Beira"[tw] or "Nampula"[tw] or "Chimoio"[tw] or "Nacala"[tw] or "Quelimane"[tw] or "Lusaka"[tw] or "Kitwe"[tw] or "Ndola"[tw] or "Kabwe"[tw] or "Copperbelt Harare"[tw] or "Bulawayo"[tw] or "Chitungwiza"[tw] or "Mutare"[tw] or "Masvingo"[tw] or "Monashonaland"[tw] or "Manicaland"[tw] 440,31416:00:48#1 Search: "Skin"[Mesh] or "Skin Care"[Mesh] or "Emollients"[Mesh] or "emollient*"[tw] or (("skin"[tw] or oil[tw] or derm*[tw] or epiderm*[tw] or massage*[tw]) AND (practice*[tw] or care[tw] or treatment*[tw] or therap*[tw] or product*[tw])) or "Massage"[Mesh] or "massage*"[tw] or "Baths"[Mesh] or "bath"[tw] or "baths"[tw] or "bathing"[tw] or "Oils"[Mesh] or "oil"[tw] or "oils"[tw] or "Aloe"[Mesh] or "aloe"[tw] or "Perinatal care"[mesh] or "postnatal care"[mesh] or "perinatal care"[tw] or "antenatal care"[tw] or "Home Care Services"[mesh] or "home care"[tw] or "Hygiene"[mesh] or hygiene[tw] 1,087,81916:00:09

### Selection of eligible studies

Title and abstract screening will be guided by a series of queries ( Table 2). Further eligibility criteria will ensure that the content of the included studies is relevant to the research question.

**Table 2.** Guiding queries used to electronically capture relevant information from each included study.

1. Author and Date 2. Title of Study 3. What was the sample size? 4. Where was the study done (city/district/province/country)? 5. Was the study done in a hospital (what level?) or home setting? 6. What was the gestational age of the neonates? 7. What was the chronological age of the neonates? 8. What substance was applied? 9. Was the substance applied to the umbilical cord? 10. How often was the substance applied? 11. By whom was the substance applied? 12. How was the substance applied? 13. How was the substance distributed on the body (i.e applied to the nappy area, the scalp, etc)? 14. What are the health impacts of product application? 15. What is the acceptability of the product to those involved in the study? 16. What product preferences do those involved in the study hold, if any? 17. Was the neonate massaged, and if so, how was the massage performed? 18. Why was this substance applied? (belief) 19. With what was the neonate bathed? 20. What was the temperature of the bath water? 21. Was anything applied to skin after bathing? (If yes, what was applied?) 22. By whom were the neonates bathed? 23. How often were the neonates bathed? 24. Why was the neonate bathed in this way (belief)? 25. What was the mode of delivery, if recorded (i.e., spontaneous vaginal delivery, cesarean section, etc)? 26. What were the outcome measures, if any? 27. What observations were made about neonates' response? 28. What descriptive norm(s), If any, influenced this scenario? 29. What injunctive norm(s), If any, influenced this scenario? 30. What perceived sanctions of norm(s), If any, influenced this scenario?

Inclusion criteria

For studies to be included, they must meet the following criteria:

- Report on skin care practices administered to newborns
- Include participants from sub-Saharan Africa
- Published after 1 January 2000 and prior to 1 July 2021
- Qualitative and quantitative studies

Exclusion criteria

Studies will be excluded if they have any of the following characteristics:

- Studies that do not include participants from Africa
- Multi-center or multi-country studies reporting data from Africa that cannot be isolated from mixed summary data that includes non-African countries
- Studies with an exclusive focus on umbilical cord care and/or bathing practices, and an absence of information on emollient applications to the skin
- Studies that are not available in English or French
- Studies where full-text of the article could not be obtained

The electronic database search will be recorded in a table (see Table 3). All eligible articles will be uploaded into the Covidence software, and duplicates identified and removed. Title and abstract screening of all eligible articles will be conducted to determine whether the study should be included in the review or not. All attempts will be made to obtain full texts of selected articles, by searching the web, engaging with librarians from University of Oxford, University of Makerere in Uganda, Busitema University Faculty of Health Science in Uganda, and Stanford University, or contacting an author if necessary. Two investigators will conduct full-text screening of the selected studies. A third reviewer will be employed if there are significant discrepancies that cannot be resolved by discussion and consensus. The degree of agreement between reviewers will be calculated and reported.

**Table 3.** Electronic database searches. An illustrative draft of the table in which electronic database searches will be recorded.

Date of search	Electronic database	Keywords searched	Number of studies retrieved	Number of studies selected
	PubMed	“neonatal massage” or “newborn massage”, AND “Africa”		

The selection process will follow the recommendations in the Preferred Reporting Items for Systematic Reviews and Meta-Analyses Extension for Scoping Reviews (PRISMA-ScR) checklist ^15^ and will be mapped using the PRISMA-P chart ^16^.

### Amendments

Important protocol amendments will be documented in future protocol files and in the narrative report of the scoping review.

### Charting the data

A data charting form will be used to electronically capture relevant information from each included study. The extracted data will include fields outlined in Table 2.

### Collating, summarizing and reporting the results

A narrative report will be produced to summarize the extracted data for the following outcomes: region (country, province) of study, prevalence of skin care-related health conditions, associated factors for skin care-related health conditions and skin care practices for newborns. The final outcome will include two sub-foci: emollient and massage. For the former, data around the following outcomes will be extracted and summarized: sample size; location of study (city/district/province/country and hospital/home setting); gestational age of neonates; chronological age of neonates; type of substance applied to the skin; whether substance was applied to the umbilical cord; how often substance was applied; by whom the substance was applied; how the substance was applied; how the substance was distributed on the body (i.e. the scalp, nappy area etc.); health impacts of product application; acceptability of the product to those involved in the study; product preferences of those involved in the study; whether the neonate was massaged, and if so, how the massage was performed; why the substance was applied; with what the neonate was bathed; temperature of the bath water; whether anything was applied to the skin after bathing, and if so, what substance; by whom the neonates were bathed; how often the neonates were bathed; why the neonate was bathed in a particular way; the mode of delivery if recorded (i.e. SVD/CS); outcome measures, if any; observations about neonate response to care; descriptive, injunctive, and/or perceived sanctions of norm(s), if any, that influenced the scenario. Regarding massage, the following data will be extracted and summarized: type of massage performed, how massage is performed, how often massage is performed, when massage is performed, who performs the massage, rationale behind the selected massage technique, perceived benefits of the technique, any harms or concerns about the technique, and health impacts of the massage technique. These results will be described in relation to the research question and in the context of the overall study purpose. Gap analysis will identify areas where data on neonatal emollient therapy are still needed in sub-Saharan Africa.

### Dissemination

The narrative report summarizing the data from the review will be disseminated in the public domain via publication in a relevant journal; circulation through the academic networks of review authors; and direct sharing to a team of researchers representing the University of Makerere in Uganda, Busitema University Faculty of Health Science in Uganda, Mbale Clinical Research Institute, Save the Children and Stanford University who are preparing for a clinical trial on neonatal emollient therapy in Uganda.

### Study status

As of 5 August 2021, we have developed and validated the ability of the search strategy to capture a set of articles of known relevance ^10^, employed the search strategy to identify eligible articles, uploaded the titles and abstracts into Covidence software, and completed an initial screening of titles and abstracts to identify articles for full-text review.

## Discussion

The proposed scoping review aims to identify and describe common newborn skin care practices throughout Africa, with a focus on bathing, the application of oils and massage. It will also highlight gaps in current knowledge on newborn skin care practices in Africa. As the quality of the studies will not be assessed, commentary cannot be provided regarding the reliability of data extracted from selected studies.

We will reflect on the implications of the findings for the feasibility and design of randomized controlled trials to examine the impacts of emollient therapy on survival, postnatal growth and neurodevelopment of very low birth weight infants in Africa. This review will support the planning of effective emollient intervention trials for newborn health in the future, particularly in sub-Saharan Africa. The evidence gaps identified by this review will also capacitate governments, funders, and researchers to refine their focus on neglected research areas and accelerate impact in newborn skin care interventions in sub-Saharan Africa. This review also has the potential to equip policy makers and stakeholders with evidence to support the care of this high-risk group of very low birth weight infants in sub-Saharan Africa.

## Data availability

No data are associated with this article.

### Reporting guidelines

Stanford Digital Repository: PRISMA-P checklist for ‘Protocol for a scoping review of neonatal emollient therapy and massage practices throughout sub-Saharan Africa’.

https://purl.stanford.edu/zx021fw5637 ^13^.

Data are available under the terms of the Creative Commons Zero "No rights reserved" data waiver (CC0 1.0 Public domain dedication).
