# Supplementary material for: Protocol for a scoping review of neonatal emollient therapy and massage practices throughout sub-Saharan Africa
Source: Gates Open Res. 2021 Aug 24;5:142. [Version 1] doi: 10.12688/gatesopenres.13359.1 (PMC8901585; doi:10.12688/gatesopenres.13359.1)
Supplement: Peer review history

# Peer review: Reviewer response for version 1

**DOI:** 10.21956/gatesopenres.14604.r31779

**Published:** 2022-03-11

**Authors:** Karen New, Deanne August

**Affiliations:** 1 School of Health and Behavioural Science, University of the Sunshine Coast, Buderim, Qld, Australia; 2 School of Nursing, Midwifery and Social Work, University of Queensland, Brisbane, Qld, Australia; 3 Grantley Stable Neonatal Unit, Royal Brisbane and Women's Hospital, Brisbane, Qld, Australia; 4 School of Medicine and Dentistry, James Cook University, Townsville, Qld, Australia

Thank you for the opportunity to provide a peer review report on your protocol for a scoping review of neonatal emollient therapy and massage practices throughout sub-Saharan Africa. The focus of the review is broad and is a large undertaking for which you are congratulated. The protocol overall articulates the scope and methods for the review, which are appropriate, however, there are some linkages missing for the reader. For example, the linkage between the rationale and review questions and outcomes. Increasing the clarity around these areas would enhance the protocol to allow replication by others.

 Presently the background/introduction discusses low birth weight and premature neonates in relation to infection and mortality linked with skin development and immature barrier function and that optimal skincare practices may offer interventions to address adverse outcomes. Additionally, emollient therapy may also promote growth, enhance neurodevelopmental outcomes and reduce hospital-acquired infection and mortality. Furthermore, cultural practices of the application of oils and other products and the effects of emollient therapy on newborn health are less known and the scoping review may address this area. The main objective of this scoping review is to create an accurate depiction of newborn skincare practices in Africa and the question addressing the objective is “what are the common newborn skincare practices throughout Africa, with a focus on bathing, the application of oils and other products, and massage?” The particular focus on bathing in this question warrants clarification in the introduction.

 The second research question “what is the reported impact of newborn skincare practices throughout Africa, for example, the impact of emollient therapy on newborn postnatal survival, growth, infection and neurodevelopment?”, does not appear to align with the main objective and perhaps there is a need for a second objective. Alternatively, the main objective could be broadened to: identify an accurate depiction **and the impact **of newborn skincare practices in Africa; including bathing, emollients and their application?

 Newborn skin care practices are vast and while the inclusion and exclusion criteria for studies provides some restrictions to what practices are being considered for this review, the background/introduction does not provide the rationale for these. There is a lack of clarity to the suggested topical solutions of interest. For example, oils (how is this defined, and does it include any oil type applied to the newborn's skin?), other products (how is this defined and does this include traditional and commercially available products?), are emollients and other moisturisers excluded? Similarly, there is a lack of linkage or explanation of the relationship between bathing and emollient and massage; and growth and oil/emollient use. For the second question, while there is some rationale for emollient therapy in preterm infants, it is not clear what is meant by emollients for this review and as the review is focusing on all newborns (not just preterm newborns), the rationale for emollient therapy for all newborns and the outcomes is lacking.

 The protocol for this review would benefit from having definitions for the key terms and outcomes stated to increase clarity for future replication and research:

- Newborn skin care practices (and to what time point. Does newborn mean neonate and therefore skincare practices within the first 28 days of life?).
- Emollient (could this term be used to describe any ointment, cream, oil and moisturisers?).
- Oil (if not included under emollient, then how is oil defined).
- Other products (to include any traditional/natural or commercial product).
- Massage (type of massage with or without the application of an emollient/oil).
- Postnatal survival (for this review it is not clear the length of time the term postnatal is referring to – this could be assumed to be 42 days post-birth, but stating this clearly would enhance the protocol/dataset at a later date).
- Growth (is this inferring weight and length, and/or head circumference) and how measured and over what time period.
- Infection (hospital-acquired only (could be very limiting to the Africa context, where homebirths occur and early discharge is common), local infection or laboratory-confirmed central infection or defined as reported in the individual studies). Over what time period.
- Neurodevelopment (time point for outcome measurement, how measured etc).

The guiding queries is a helpful section for the reader, much of which is clearly articulated and of relevance for this broad topic. However, there is the potential to better align some of the content of these guiding queries to the outcomes in the review questions. For example, Questions 19-24 appear focused on bathing (which links to the need for more rationale around bathing and the link with emollient therapy and massage practices) and cultural context. If the aim is to assess emollient practices including in the event of bathing that is fine, but this should be more related to the timing between application and post bathing. Q21 is a little ambiguous in asking was anything applied to the skin after bathing, which alludes to bathing and the application of a product that may be relevant to emollient therapy and/or massage, but this is not clear. Furthermore, Question 8 and the use of the term ‘substance’ is a little confusing and in this context does ‘substance’ substitute for oil, emollient and any other product? While the frequency of the application of the substance is being captured, the volume/amount is not which may also be important for outcomes. Further clarity around these guiding queries would enhance the ability for replication by others.

 Regarding the second research question and the impact of emollient therapy on survival, growth, infection and neurodevelopment – the guiding queries do not appear to be directed at these outcomes. Although Question 14 asks about the health impacts it is not clear what is being captured by this question for the purpose of addressing the review questions or objective.  

 The search timeframe of 20 years would benefit from a rationale/justification. 20 years is a relatively long timeframe if the intent of the review is to determine contemporary practices. However, the timeframe may be very relevant if looking for changes over time or from when the first international Neonatal Guideline was published. Such a rationale would again increase the understanding of why this was done. This latest version of the protocol does not appear to have added the CINAHL database to be searched, a suggestion from the first reviewer, in which we agree as this would be an important database to capture top nursing literature which would be very relevant to the objective and questions for this scoping review. 

 The current title of the scoping review implies the review is focused on emollient therapy and massage as opposed to aligning with the main objective which appears to be creating a depiction and the impact of newborn skincare practices in sub-Saharan Africa. Thus, a review of the title to reflect the objective of the review more accurately may be warranted.

 The undertaking of this scoping review is important and will be valuable in identifying gaps in the evidence and therefore future research needs. We hope our feedback on this review protocol assists in increasing the clarity of the review in order to enhance the reporting of outcomes and datasets.

Is the study design appropriate for the research question?

Yes

Is the rationale for, and objectives of, the study clearly described?

Partly

Are sufficient details of the methods provided to allow replication by others?

Partly

Are the datasets clearly presented in a useable and accessible format?

Partly

Reviewer Expertise:

Neonatology, neonatal nursing, neonatal skin development, injury and skincare, scoping review methods, qualitative and quantitative research methods.

We confirm that we have read this submission and believe that we have an appropriate level of expertise to confirm that it is of an acceptable scientific standard, however we have significant reservations, as outlined above.

# Peer review: Reviewer response for version 1

**DOI:** 10.21956/gatesopenres.14604.r31781

**Published:** 2022-03-07

**Authors:** Albert Manasyan

**Affiliations:** 1 University of Alabama at Birmingham, Birmingham, AL, USA; 2 Centre for Infectious Disease Research in Zambia, Lusaka, Zambia

The protocol addresses a research question that may contribute to one of the top 3 contributors to neonatal mortality. I have only the following 2 comments/suggestions:

1. It may be interesting to understand if the location of birth impacts such practices - home deliveries having a higher chance of such practices versus facility deliveries. If this is of interest to the Investigators, this can be added to Table 2.
2. For the second objective ("...reported impact of newborn care practices"), would the investigators be able to review clinical data (i.e., Abx treatment) and KMC practices when evaluating the findings?

Is the study design appropriate for the research question?

Yes

Is the rationale for, and objectives of, the study clearly described?

Yes

Are sufficient details of the methods provided to allow replication by others?

Yes

Are the datasets clearly presented in a useable and accessible format?

Yes

Reviewer Expertise:

Perinatal research, neonatal mortality, LMIC settings, primarily SSA).

I confirm that I have read this submission and believe that I have an appropriate level of expertise to confirm that it is of an acceptable scientific standard.

# Peer review: Reviewer response for version 1

**DOI:** 10.21956/gatesopenres.14604.r31138

**Published:** 2021-09-02

**Authors:** Alison W. Talbert

**Affiliations:** 1 KEMRI-Wellcome Trust Research Programme, Kilifi, Kenya

The protocol addresses a topic that has been chosen for future research in sub-Saharan Africa. The rationale and methods for the proposed scoping review are well described and I have only minor comments to make:

- On search strategy: have you considered searching CINAHL (Cumulative Index to Nursing and Allied Health Literature) database? There may be some relevant publications there.
- Remember to include details of studies excluded after full text review with reasons for exclusion as an appendix to the final review

Is the study design appropriate for the research question?

Yes

Is the rationale for, and objectives of, the study clearly described?

Yes

Are sufficient details of the methods provided to allow replication by others?

Yes

Are the datasets clearly presented in a useable and accessible format?

Not applicable

Reviewer Expertise:

Paediatrics, epidemiology, nutrition

I confirm that I have read this submission and believe that I have an appropriate level of expertise to confirm that it is of an acceptable scientific standard.
